# Total and segmental phase angle in a cohort of hospitalised patients with COVID-19: mortality prediction and changes throughout hospitalisation

**DOI:** 10.1017/S0007114523002994

**Published:** 2024-04-28

**Authors:** Fernando Estrada-Moya, Alejandro G. González-Garay, Adriana Flores-López, Aurora E. Serralde-Zúñiga

**Affiliations:** 1 Servicio de Nutriología Clínica, Instituto Nacional de Ciencias Médicas y Nutrición Salvador Zubirán, Mexico City, Mexico; 2 Sección de Estudios de Posgrado e Investigación, Instituto Politécnico Nacional “Escuela Superior de Medicina”, Mexico City, Mexico; 3 Departamento de Metodología de la Investigación, Instituto Nacional de Pediatría, Mexico City, Mexico; 4 Facultad de Medicina, Universidad Nacional Autónoma de México, Mexico City, Mexico

**Keywords:** Bioelectrical impedance analysis, COVID-19, Phase angle, SARS-CoV-2, Survival rate, Bioelectrical impedance vector analysis, Segmental analysis

## Abstract

Body composition and phase angle (PhA) have been used to predict mortality in multiple diseases. However, little has been studied regarding segmental measurements, which could potentially help assess subtle changes in specific tissue segments. This study aimed to identify the total PhA cut-off point associated with mortality risk and changes in body composition within a week of hospitalisation in non-critical hospitalised patients with COVID-19. A cohort study was conducted where patients underwent to a complete nutritional assessment upon admission and after seven days, and followed up until hospital discharge or death. A receiver operating characteristic curve was constructed to determine the PhA cut-off point, and the Kaplan–Meier estimator was used to determine survival analysis. Segmental and complete body compositions on admission and after 7 d were compared. We included 110 patients (60 men) with a mean age of 50·5 ± 15·0 years and a median BMI of 28·5 (IQR, 25·6–33·5) kg/m^2^. The median length of hospital stay was 6 (IQR, 4–9) d, and the mortality rate was 13·6 %. The PhA cut-off point obtained was 4°, with significant differences in the survival rate (*P* < 0·001) and mortality (HR = 5·81, 95 % CI: 1·80, 18·67, *P* = 0·003). Segmental and whole-body compositions were negatively affected within one week of hospitalisation, with changes in the approach by the graphical method in both sexes. Nutritional status deteriorates within a week of hospitalisation. PhA < 4° is strongly associated with increased mortality in non-critical hospitalised patients with COVID-19.

## Graphical abstract

**Figure:**
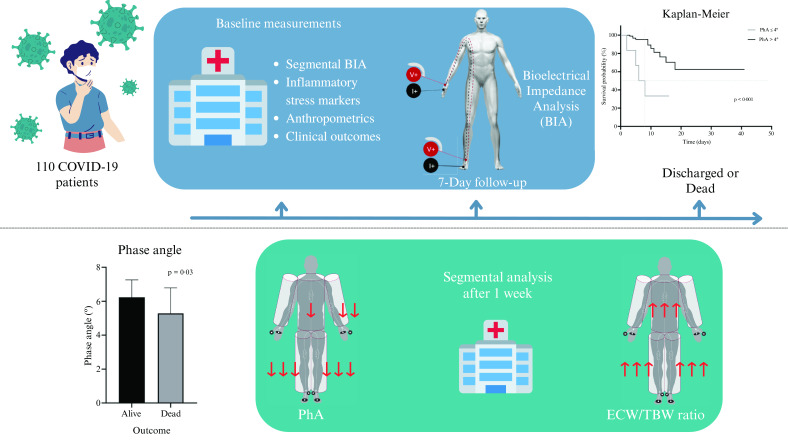

The coronavirus disease (COVID-19), caused by severe acute respiratory syndrome coronavirus 2 (SARS-CoV-2), was first described in January 2020^(1,2)^. In March 2020, the World Health Organization (WHO) declared the COVID-19 outbreak to be a pandemic^(3)^. Recently, the WHO declared that COVID-19 no longer constitutes an international public health emergency; however, it will continue to be an established and persistent health problem. Therefore, research must continue to improve our understanding of and approach towards this disease and develop the necessary tools to face future pandemics^(4)^.

The clinical spectrum of COVID-19 can range from asymptomatic infection to severe illness and even death^(5,6)^, of patients who develop symptoms; approximately 20 % require hospitalisation, 4·9 to 11·5 % require invasive mechanical ventilation^(5)^, and the fatality rate ranges from 3·4 to 20 %, depending on the risk factors of the patients^(6)^. Mortality associated with poor nutritional status has been reported in this pathology^(7)^, with a particularly high nutritional risk associated with multifactorial aetiology. Male sex, high blood pressure, diabetes mellitus, cardiovascular disorders, advanced age, D-dimer values > 1 μg/ml, and a high Sequential Organ Failure Assessment score have been associated with a high case fatality rate^(8)^. Numerous studies have shown that malnutrition can influence patient clinical outcomes, including mortality. Patients with high nutritional risks showed a higher frequency of mortality than patients with low nutritional risk (50·8 % *v*. 40 %; *P* = 0·014). The probability of death almost doubled, regardless of the presence of other comorbidities (HR = 1·74; *P* < 0·001)^(9)^.

Nutritional assessment, especially body composition, benefits from the use of bioelectrical impedance analysis (BIA), given its ease of use, safety, portability, and affordability compared to reference standards such as computed tomography (CT) scan and dual X-ray absorptiometry. It is based on impedance measurements and is composed of two elements, resistance (R) and reactance (Xc), using one or multiple electrical frequencies, showing correlation coefficients ranging from 0·74 to 0·98 when compared to the reference standards^(10,11)^. These data provide additional information beyond whole-body measurements as they provide segmental data that can potentially help to assess subtle changes in specific tissue segments^(12)^.

Phase angle (PhA) is a cell integrity indicator that is widely used as a prognostic predictor and is integral to the nutritional assessment of patients with multiple diseases, including hepatic, renal, and oncological diseases^(13)^. PhA is calculated by taking the values of R and Xc with the following formula: PhA = arctan (Xc/R) × 180π^(11)^. The results are obtained in degrees (°) and interpreted according to the reference values per population. In the last few years, the bioelectrical impedance vector analysis (BIVA), also called the graphical R/Xc (RXc) method, has been used mainly in healthy athlete populations but also in some clinical situations^(14–17)^. This method uses height to standardise R and Xc (R/height and Xc/height, respectively), which are components of the impedance vector on an RXc graph, to compare the individual’s measurement to that of a healthy population. In contrast to the commonly used body composition analysis where fat is the primary component identified, this method also includes hydration and muscle mass assessment, independent of the individual’s body weight^(18–20)^. This study aimed to determine the PhA cut-off point associated with a higher risk of mortality in non-critically hospitalised patients with COVID-19 and to determine the changes in body composition within a week of hospitalisation. We hypothesised that a low PhA would be associated with increased mortality risk in non-critical hospitalised patients with COVID-19.

## Methods

### Study design and participants

This cohort study was conducted at a non-critical area of a tertiary care centre from January to December 2021. We included admitted patients of both sexes aged ≥ 18 years with a reverse transcription polymerase chain reaction (RT-PCR)-confirmed COVID-19 diagnosis. Pregnant or lactating women and those with pacemakers, metal plates, amputations, or extensive skin lesions in the electrode placement area were excluded. Patients who were sent to another hospital unit for management and could not complete the measurements were excluded from the study.

Data on all independent variables, including demographic, biochemical, and clinical information on pneumonia severity by CT^(21)^, were obtained from the patients’ electronic clinical files; nutritional assessment variables were obtained directly from the patients and their families within 24 h of hospital admission. For recording the dependent variables, length of stay (LOS) and mortality, the enrolled patients were followed up daily until they were discharged or died. This study was conducted according to the guidelines laid down in the Declaration of Helsinki, and all procedures involving humans were approved by the Institutional Review Board (Comité de Investigación and Comité de Ética en Investigación from Instituto Nacional de Ciencias Médicas y Nutrición Salvador Zubirán); reference number 3333. Verbal informed consent was witnessed and formally recorded. No harm was caused to the patients, and participant data were anonymized and kept confidential.

### Nutritional assessment

Weights were obtained by Lohman’s methodology^(22)^ using the Seca® 954 scale. Dynamometry was performed using the Jamar® hydraulic hand dynamometer in the patient’s dominant hand, registering the higher of the three measurements. Mid-arm and calf circumferences were measured with the Lufkin® anthropometric tape (Executive Thinline) according to the technique described by ISAK^(23)^.

We asked patients and their families about their current percentage of food intake compared to their usual diet (before they got sick) and registered this information as a clinical independent variable.

BIA measurements: raw data (R and Xc), total and segmental body composition data, including PhA, were obtained using the InBody® S10 body composition analyser (Inbody Co., Ltd., Seoul, Korea). All measurements were performed by trained staff according to the following standardised technique: the patient (fasting and with an empty bladder) was instructed to lie in a supine position; electrodes were placed on the patient’s cleaned hands and feet; the identifier number, age, height, and gender were recorded in the equipment; and, the measurements were obtained after a lapse of 3 min, in raw and printable form.

A second nutritional assessment was conducted in patients with ≥ 1 week of LOS. We performed a secondary analysis to compare the changes after 7 d of hospitalisation with data recorded at baseline. The hydration levels, extracellular water/total body water (ECW/TBW) ratio, and PhA at a frequency of 50 kHz were reported segmentally (right arm, left arm, trunk, right leg, and left leg). In this secondary analysis, the impedance vector was determined using the BIVA method.

### Statistical analysis

Regarding the descriptive analysis of the variables, for quantitative variables, the Kolmogorov–Smirnov test was conducted to assess their normality. Variables with a normal distribution were reported as mean and standard deviation, while those with a non-normal distribution were presented as the median and interquartile range (IQR).

The qualitative variables are presented as frequencies and percentages. In the bivariate inferential analysis, quantitative variables with parametric distribution were analysed using Student’s t-test, whereas the quantitative variables with a non-normal distribution were analysed using the Mann–Whitney U test, and the qualitative variables were analysed using the Chi-square test or Fisher exact test.

To determine the cut-off point for PhA, a receiver operating characteristic (ROC) curve analysis was performed. Subsequently, an analysis was conducted to know the potential confounding variables, using the Mann–Whitney U test for quantitative variables and the Fisher exact test for qualitative variables. A survival analysis (considering the cut–off point obtained from the ROC curve) using the Kaplan–Meier estimator was made. Differences in the curves were analysed using the log-rank test. Finally, a Cox Regression was used to determine the hazard ratio (HR) to measure the association to report the risk; all these analyses were performed adjusting by cofounders. In patients with a LOS ≥ 1 week, a secondary analysis was carried out, and quantitative variables with a parametric distribution were analysed using the paired t-test. Quantitative variables with a non-normal distribution were analysed using the Wilcoxon signed-rank test. The impedance vector was determined by the BIVA method. Finally, we analysed the relationship between segmental changes (PhA and ECW/TBW) and prognosis using a Cox regression and random-effects parametric survival model.

Statistical significance was set at *P* < 0·05. Statistical analyses were performed using the STATA statistical software (StataCorp LLC Version 15.1), the GraphPad Prism software (GraphPad Software, Inc., version 8) was used for graphing, and BIVA software (2002 BIVA tolerance file; Antonio Piccoli) was used for the RXc graphic method.

The sample size was calculated before the participants were included in the survival study^(24)^; considering a statistical power of 80 % (*β* = 0·842) and a level of confidence of 95 % (*α* = 1·96), referring to the values of relative risk (2·48) and the proportion of exposed patients (0·41) in the article published by Cornejo et al.^(25)^, and considering possible losses of 20 %, we obtained a sample size of 110.

## Results

**Fig. 1.**
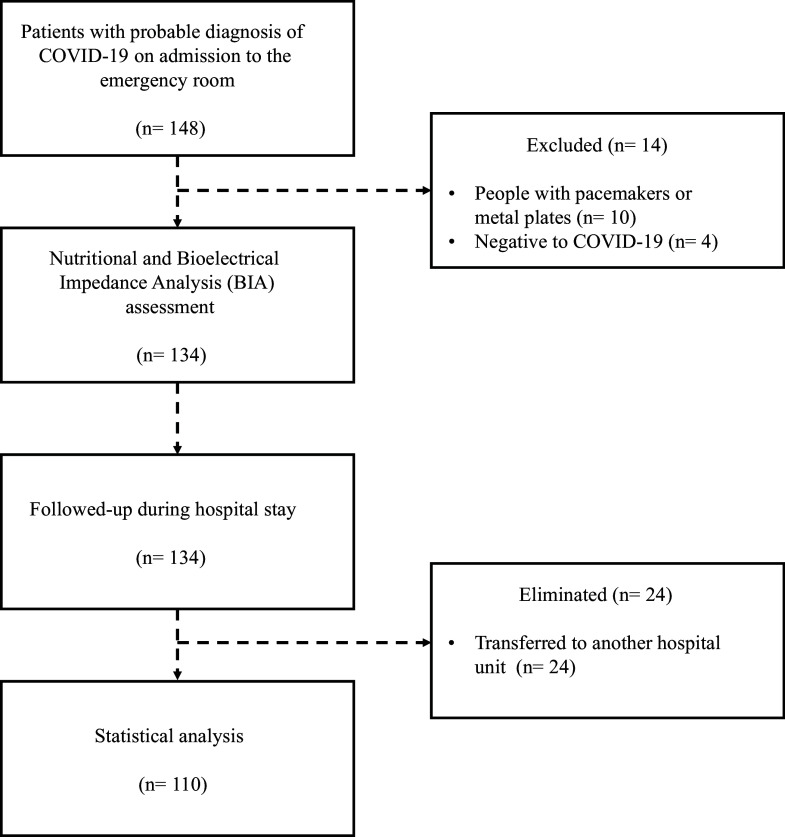
Patient selection flowchart.

In total, 110 patients were included. Figure 1 shows the selection process flowchart; 54·5 % of the patients were men, the mean age of the patients was 50·5 ***±*** 15·0 years, and the median BMI was 28·5 kg/m^2^ (IQR, 25·6–33·5). Table 1 displays the anthropometric and BIA differences found between men and women, as well as the baseline characteristics of the patients: 60 % had severe pneumonia, 35·5 % had moderate pneumonia, and 4·5 % had mild pneumonia. The weight loss rate from the onset of symptoms until hospital discharge was 3·4 % (IQR, 0·5–6·3). The median percentage of food intake compared with the usual diet was 70 %.

**Table 1.** Baseline characteristics

	All (*n* 110)				
	Median	IQR			
Inflammatory stress markers					
C-Reactive Protein (mg/dl)					
Mean	12·9				
sd	7·9				
Ferritin (ng/ml)*	432	215–810			
D-Dimer (ng/ml)*	682	485–1168			
Albumin (g/dl)					
Mean	3·7				
sd	0·4				
Respiratory					
PaO_2_/FiO_2_ score*,†	236	173–301			
	*n*	%			
Comorbidities					
Diabetes mellitus	35	31·8			
Hypertension	40	36·4			
Chronic obstructive pulmonary disease	5	4·50			
Cardiovascular disease	5	4·50			
Chronic kidney disease	11	10·0			
Liver disease	7	6·4			
Immunosuppression	13	11·8			
Cancer	5	4·5			
	**Women (*n* 50)**		**Men (*n* 60)**		***P* value**
	**Median**	**IQR/**	**Median**	**IQR**	
Anthropometrics					
Height (cm)					
Mean	156·4		170·7		0·000
sd	7·6		7·3		
Actual body weight (kg)*	70·9	60·7–86	85·9	70·4–98·9	0·066
Weight loss (%)*	3·8	1·9–7	2·8	0·4–5·5	0·282
Calf circumference (cm)*	36·8	33–39·5	37·3	34·5–39·8	0·986
Mid-arm circumference (cm)*	32	28·5–36	33	29·6–35·2	0·338
Handgrip strength (kg)					
Mean	20·7		36·7		0·000
sd	7·1		10·7		
Bioelectrical impedance analysis					
Phase angle (º)					
Mean	5·6		6·5		0·000
sd	1·0		1·1		
Skeletal muscle mass (%)*	33·4	30·9–36·1	42·6	38·9–46·8	0·000
Body fat mass (%)					
Mean	38·7		28·1		0·000
sd	9·9		9·6		
Skeletal muscle mass index (kg/m^2^)*	7·6	6·5–8·1	8·8	8·2–9·6	0·000
Visceral fat area (cm^2^)*	132·1	82·7–171·4	87·1	59·7–127·2	0·004
Extracellular water/Total body water					
Mean	0·381		0·376		0·043
sd	0·013		0·013		

The data are presented as the mean ± standard deviation or median with interquartile range (25th–75th percentile) depending on the normalcy of the data distribution.

* The differences between the groups at baseline were evaluated using Student’s *t* test or the Mann–Whitney U test (*) for continuous variables, and proportions were compared using the Chi^2^ or Fisher test.

† PaO^2^/FiO^2^ score: partial pressure arterial oxygen/fraction of inspired oxygen score.

The mortality rate in the sample was 13·6 %, with no differences in sex (six women *v*. nine men; *P* = 0·783), pneumonia severity, and PaO_2_/FiO_2_ (partial pressure arterial oxygen/fraction of inspired oxygen). However, the age was lower in the group of patients who survived than in those who died (49·2 ***±*** 14·7 *v*. 59·3 ± 14·5 years; *P* = 0·021).

The initial data show a higher proportion of death in patients who had a previous diagnosis of diabetes mellitus (26 (27·4 %) *v*. 9 (60 %); *P* = 0·012), hypertension (31 (32·6 %) *v*. 9 (60 %); *P* = 0·041)) and chronic kidney disease (7 (7·4 %) *v*. 4 (26·7 %); *P* = 0·042) upon admission, without showing differences in the proportions of patients with chronic obstructive pulmonary disease, cardiovascular disease, liver disease, immunosuppression, and cancer. However, after analysing the data according to the PhA cut-off point, there was no difference in the frequency of comorbidities in both groups, except for chronic kidney disease, which shows a higher proportion in the group of patients with a PhA < 4° (7·7 % *v*. 50 %; *P* = 0·013).

The handgrip strength was significantly lower (30·4 ***±*** 12·1 *v*. 23·2 ***±*** 10·9 kg; *P* = 0·029) in the patients who died. In the BIA, the PhA was significantly lower (6·2 ± 1·0 *v*. 5·3 ± 1·5 º; *P* = 0·032), and the ECW/TBW ratio was higher (0·377 ± 0·012 *v*. 0·389 ***±*** 0·018; *P* = 0·032) in the group of patients who died; mortality was negatively correlated with albumin level (R^2^ = −0·504; *P* < 0·001), it being the only biochemical marker with lower levels in the patients who died.

**Fig. 2.**
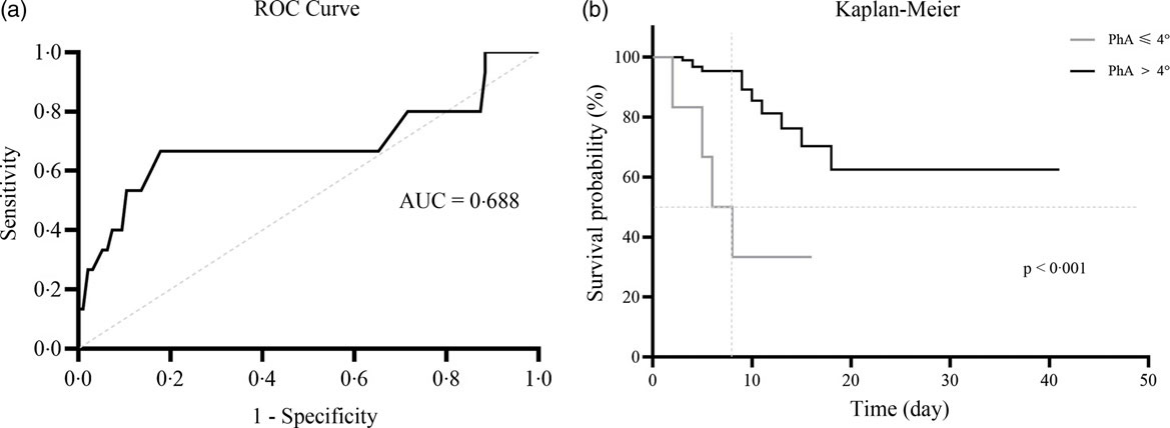
(a) Phase angle (PhA) receiver operating characteristic (ROC) curve. The ROC curve analysis determined that the cut-off point of PhA of 4° presented an area under the curve of 0·688 (95 % CI 0·506, 0·739), with a sensitivity of 26·67 % and specificity of 97·89 % and an ability to classify 88·18 % of the patients correctly. (b) Kaplan–Meier survival plot by phase angle. Log-rank test of equality analysis was performed; a significantly lower survival rate was found in the group with a PhA ≤ 4° (*P* < 0·001) compared to those patients who presented a PhA > 4°.

The ROC curve analysis (Fig. 2(a)) showed that the PhA cut-off point of 4° had the highest area under the curve. When determining potential confounding variables, a statistically significant difference was found in diabetes mellitus (25 (26·9 %) *v*. 8 (72·7 %); *P* = 0·004), and in systemic arterial hypertension (30 (32·3 %) *v*. 7 (63·6 %); *P* = 0·05); all analyses were adjusted for both confounding variables. We found a significantly lower survival percentage in the patients with PhA ≤ 4° compared to those who presented PhA > 4° (Fig. 2(b); *P* < 0·001). PhA ≤ 4° was strongly associated with mortality (HR = 5·81, 95 % CI 1·80, 18·67; *P* = 0·003), adjusted for diabetes mellitus and systemic arterial hypertension.

The secondary analysis carried out in 34 patients allowed us to observe the changes within a week of hospitalisation, where decreases in weight (81·5 kg (IQR, 67·8–105·8) *v*. 77·9 kg (IQR, 64·8–103·3); *P* < 0·001), BMI (31·5 ± 8·1 *v*. 29·9 ± 7·8 kg/m^2^; *P* < 0·001), calf circumference (37·1 ± 4·4 *v*. 35·8 ± 4·4 cm; *P* < 0·001), mid-arm circumference (33·9 ± 5·9 *v*. 32·8 ± 6·2 cm; *P* < 0·001), handgrip strength (29 ***±*** 11·0 *v*. 22·8 ± 11·3 kg; *P* < 0·001), PhA (5·96 ± 1·13 *v*. 5·34 ***±*** 0·94°; *P* < 0·001), and albumin level (3·6 ± 0·4 *v*. 3·2 ± 0·4 g/dl; *P* < 0·001) were observed.

**Fig. 3.**
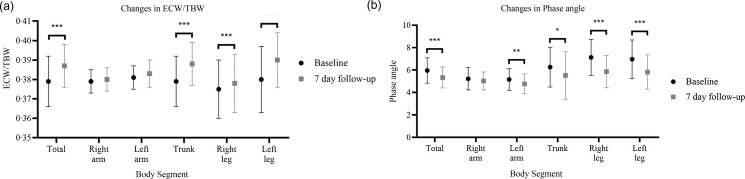
(a) Changes in extracellular water/total body water (ECW/TBW) ratio after one week of follow-up. (b) Changes in phase angle (PhA) after one week of follow-up. Analysis was performed using a paired t-test, where *P < 0·05, **P < 0·01 and *** *P* < 0·001.

Conversely, the total levels in the ECW/TBW ratio (0·379 ***±*** 0·013 *v*. 0·387 ± 0·011; *P* < 0·001) showed a significant increase after one week of hospitalisation. In Fig. 3, the segmental analysis of both the ECW/TBW ratio and PhA at 50 kHz shows the changes within a week of hospitalisation, with significant differences in the ECW/TBW ratio (Fig. 3(a)) in the trunk, right leg, and left leg and significant differences in the PhA (Fig. 3(b)) in the left arm, trunk, right leg, and left leg.

**Fig. 4.**
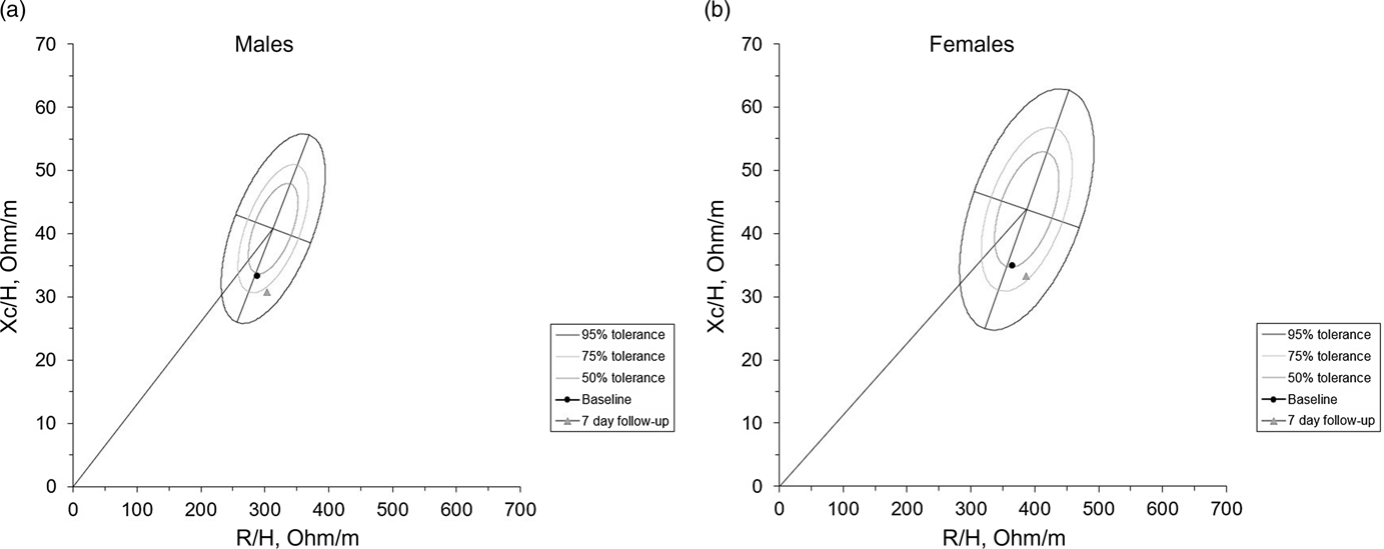
(a) Impedance vector changes (graphical method (RXc)). The images show the changes in resistance (R) and reactance (Xc), standardised by height (H) measured in ohms/metre after one week of hospitalisation. (a) Changes in men, (b) Changes in women.

Figure 4 shows the changes that occurred through the impedance vector with the BIVA method after seven days of hospitalisation, where an increase on the hydration axis can be observed, as well as a decrease in the body tissue axis with changes in R (532·1 ± 78·5 *v*. 559·6 ± 106·1; *P* = 0·02); Xc (55·2 ± 10·8 *v*. 51·9 ± 11·0; *P* = 0·04) and impedance (535·1 ± 78·5 *v*. 562·1 ± 106·3; *P* = 0·02).

When analysing the relationship between BIA segmental changes (PhA and ECW/TBW) and prognosis, using a Cox regression and random-effects parametric survival model, we did not find any statistically significant differences.

## Discussion

Nutritional assessments in patients with COVID-19 must be personalised and adapted to the needs and capacities of healthcare institutions^(5)^. Weight loss in patients with COVID-19 may be associated with reduced food intake and hypercatabolism^(26)^. The albumin levels were lower in the group of patients who died, with a negative correlation with the ECW/TBW ratio, as expected, as the decrease in the albumin level leads to a reduction in oncotic pressure. This can clinically manifest as oedema, which complicates nutritional assessment in hospitalised patients, as measurements such as weight and circumference lack precision due to fluid overload^(27)^.

The degree of malnutrition is strongly associated with the PhA, even in circumstances where most assessment tools fail because of the wrong impression that total body weight (including excess fluid retention) reflects the real amount of lean mass^(28)^. Mortality did not significantly differ based on pneumonia severity by chest CT scan in the bivariate analysis. Although this imaging technique has been reported to help stratify the severity of lung lesions and predict the prognosis, in this cohort, other factors played more significant roles in the mortality of patients with COVID-19, regardless of the percentage of alveolar occupancy found in the CT scan. We also observed that age is a critical factor affecting mortality in patients with COVID-19, similar to what has been reported in the literature^(6)^; however, higher mortality in men than in women^(2)^ was not documented in this study, perhaps due to the sample size or area around the hospital in which the patients were recruited.

In contrast, the PhA showed differences with respect to mortality, as demonstrated earlier. Garlini et al. showed in a systematic review that the PhA could be a good prognostic marker of mortality in multiple pathologies such as cancer, heart failure, kidney disease, and human immunodeficiency virus infection^(29)^. Similarly, a recent systematic review showed that patients with COVID-19 with lower PhA values had deteriorating clinical conditions, demonstrating the potential of PhA as a predictor of unfavourable clinical outcomes^(30)^.

Besides the PhA, dynamometry also showed significant differences when analysing mortality; this was expected, as dynamometry indicates the patient’s functionality and the quantity and quality of muscle mass^(31)^. Therefore, it should be considered in the nutritional assessment as it provides clinically relevant information and is an inexpensive method. The PhA cut-off point obtained was 4°, with greater sensitivity and specificity.

This value is very similar to that found by Cornejo et al. in a Spanish cohort of patients with COVID-19; low PhA values (< 3·95°) indicated a very high risk of poor prognosis and mortality. They also mentioned that the PhA offers greater sensitivity as a predictive test for prognosis on admission compared to the established analytical parameters of poor prognosis (e.g. C-reactive protein, lymphocytes, and prealbumin)^(25)^. The survival analysis confirmed that the difference in mortality, according to the PhA cut-off point of 4°, adjusted by confounding variables, was statistically significant; such results have also been seen in populations with critical COVID-19, where a higher risk of dying was associated with decreased PhA values (with HR = 5·88, *P* = 0·02)^(32)^.

Because the PhA measurement is quick and easy to obtain, it can be a valuable clinical parameter to assess the risk of severe course of disease^(33)^. At hospital discharge, there is a lower percentage of swallowing recovery in extubated patients who presented with a decreased PhA (< 4·8°)^(34)^. As PhA is an indicator obtained directly from BIA and is not subject to mathematical modelling, it is a crude measure with a good prognostic capacity; the greater the number of cell membranes the signal has to pass through, the greater the reactance, and therefore, the PhA. Thus, a large PhA is consistent with a large body cell mass^(11)^. It can be considered useful for tracking individuals at nutritional risk. Furthermore, decreased PhA values and mortality showed strong associations^(29,32)^.

Changes in body composition observed in the secondary analysis in which the nutritional assessment was performed at baseline and after 7 d of hospitalisation allowed us to understand the particularly increased nutritional risk characteristics of patients with COVID-19^(35)^. All measurements in these patients were negatively affected, both in anthropometric and biochemical variables and also in body composition. Besides being explained by persistent catabolism^(36)^, these changes are related to symptoms that make oral feeding difficult for patients, particularly due to anorexia generated by systemic inflammation. We did not find any statistically significance difference when investigating the relationship between segmental changes of PhA and ECW/TBW with mortality. This can be easily explained since only 30 % of the sample were followed up, including those patients with a LOS ≥ 1 week.

The strength of this study is that there was little evidence of segmental changes in body composition. However, in previous studies, fluid accumulation in the trunk increased by 63 % during hospitalisation after abdominal surgery, whereas it increased by 8 % and 11 % in the arms and legs, respectively. Before thoracic surgery, fluid accumulation was 93 % in the trunk and approximately 2 % in the arms and legs^(36)^. In patients with COVID-19, alterations in PhA and fluid retention in all body segments have also been observed when comparing patients with dysphagia with those without it after being extubated by COVID-19^(34)^. Although our patients did not undergo a state of stress due to surgery, COVID-19 generates significant systemic stress; therefore, the previously mentioned results were comparable with those of the present study, where, in the segmental analysis, we observed an increase in the ECW/TBW ratio in the trunk and lower body extremities, as well as a decrease in the PhA in the left arm, trunk, and lower body extremities, besides changes in their total values.

The BIVA method showed a shift in the position within a week of hospitalisation in both men and women. This can be interpreted as a decrease in body muscle mass and an increase in the patient’s fluid levels, which have been associated with functional deterioration and an increased risk of various clinical outcomes, including mortality, as Cornejo-Pareja et al., and Samoni et al. found. Cornejo-Pareja et al. showed that in patients with COVID-19, overhydration is related to mortality risk after ninety days^(37)^ and Samoni et al. reported that in patients from the ICU, the changes in body fluids to overhydration after five days also lead to higher mortality risk^(38)^. This can be explained because overhydration is the result of an imbalance where extracellular water is increased more than intracellular water; when this occurs, the balance of electrolytes, proteins, and other cellular compounds may be disrupted, leading to potential alterations in cell functions within the body; this is why several authors have linked this imbalance to a negative prognosis^(39)^.

One of the limitations of this study was that the patients were only from non-critical areas. The study sample was medium-sized, implying that more scientific evidence is required. Moreover, the volume of data obtained was not sufficient to determine the PhA cut-off point according to sex since the total mortality frequency found in this population was 15 patients. Given such a low frequency, a statistically significant cut-off point could not be determined for each sex, even though it has already been established that PhA values tend to be higher in men. Another limitation was that at the beginning of the recruitment of the study participants, SARS-CoV-2 vaccines were not yet available; however, they began to be applied during the study, and this information was not recorded. Therefore, the analysis did not differentiate between those with or without the vaccine, type of vaccine administered, or time since the vaccine was administered, which may influence the interpretation of the observed results.

### Conclusion

Regardless of the severity of COVID-19-acquired pneumonia, a low PhA (≤ 4°) was strongly associated with increased mortality in non-critical hospitalised patients. Therefore, adding this measurement as an integral part of the nutritional assessment and as a predictor of mortality in hospitalised patients with COVID-19 is recommended. Segmental analysis and the RXc graphical method can also be considered for these patients, particularly in those with fluid disorders. Nutritional assessment and reassessment of patients with COVID-19 should be performed constantly because of the alterations they present within a week of hospitalisation that represent the increasing risk of adverse clinical outcomes.

## Supporting information

Estrada-Moya et al. supplementary material Estrada-Moya et al. supplementary material

## Supplementary material

For supplementary material/s referred to in this article, please visit https://doi.org/10.1017/S0007114523002994

## References

[1] Atzrodt CL , Maknojia I , McCarthy R , et al. (2020) A guide to COVID-19: a global pandemic caused by the novel coronavirus SARS-CoV-2. FEBS J 287, 3633–3650. 32446285 10.1111/febs.15375 PMC7283703

[2] Jin Y , Yang H , Ji W , et al. (2020) Virology, epidemiology, pathogenesis, and control of COVID-19. Viruses 12, 372. 32230900 10.3390/v12040372 PMC7232198

[3] WHO Director-General’s (2020) WHO. Coronavirus Disease (COVID-2019) Situation Report-51. (Internet). https://www.who.int/docs/default-source/coronaviruse/situation-reports/20200311-sitrep-51-covid-19.pdf?sfvrsn=1ba62e57_10 (accessed 10 November 2021).

[4] WHO Director-General (2023) WHO, Statement. Statement on the Fifteenth Meeting of the IHR (2005) Emergency Committee on the COVID-19 Pandemic. https://www.who.int/news/item/05–05–2023-statement-on-the-fifteenth-meeting-of-the-international-health-regulations-(2005)-emergency-committee-regarding-the-coronavirus-disease-(covid-19)-pandemic (accessed 18 May 2023).

[5] Minnelli N , Gibbs L , Larrivee J , et al. (2020) Challenges of maintaining optimal nutrition status in COVID-19 patients in intensive care settings. J Parenteral Enteral Nutr 44, 1439–1446. 10.1002/jpen.1996 PMC7461277 32799322

[6] Onder G , Rezza G & Brusaferro S (2020) Case-fatality rate and characteristics of patients dying in relation to COVID-19 in Italy. JAMA 323, 1775–1776. 32203977 10.1001/jama.2020.4683

[7] Gonzalez MC (2019) Using bioelectrical impedance analysis for body composition assessment: sorting out some misunderstandings. J Parenteral Enteral Nutr 43, 954–955. 10.1002/jpen.1702 31486082

[8] Zhou F , Yu T , Du R , et al. (2020) Clinical course and risk factors for mortality of adult inpatients with COVID-19 in Wuhan, China: a retrospective cohort study. Lancet 395, 1054–1062. 32171076 10.1016/S0140-6736(20)30566-3 PMC7270627

[9] Palermo dos Santos AC , Japur CC , Passos CR , et al. (2022) Nutritional risk, not obesity, is associated with mortality in critically ill COVID-19 patients. Obes Res Clin Pract 16, 379–385. 36041995 10.1016/j.orcp.2022.08.005 PMC9395293

[10] Lukaski HC , Kyle UG & Kondrup J (2017) Assessment of adult malnutrition and prognosis with bioelectrical impedance analysis. Curr Opin Clin Nutr Metab Care 20, 330–339. 28548972 10.1097/MCO.0000000000000387

[11] Moonen HPFX & Van Zanten ARH (2021) Bioelectric impedance analysis for body composition measurement and other potential clinical applications in critical illness. Curr Opin Crit Care 27, 344–353. 33967207 10.1097/MCC.0000000000000840 PMC8270506

[12] Tinsley GM , Harty PS , Moore ML , et al. (2019) Changes in total and segmental bioelectrical resistance are correlated with whole-body and segmental changes in lean soft tissue following a resistance training intervention. J Int Soc Sports Nutr 16, 58. 31783760 10.1186/s12970-019-0325-4 PMC6883592

[13] Moonen HPFX , van Zanten FJL , Driessen L , et al. (2021) Association of bioelectric impedance analysis body composition and disease severity in COVID-19 hospital ward and ICU patients: the BIAC-19 study. Clin Nutr 40, 2328–2336. 33129597 10.1016/j.clnu.2020.10.023 PMC7577288

[14] Nwosu AC , Mayland CR , Mason S , et al. (2019) Bioelectrical impedance vector analysis (BIVA) as a method to compare body composition differences according to cancer stage and type. Clin Nutr ESPEN 30, 59–66. 30904230 10.1016/j.clnesp.2019.02.006

[15] Martins PC , Gobbo LA & Silva DAS (2021) Bioelectrical impedance vector analysis (BIVA) in university athletes. J Int Soc Sports Nutr 18, 7. 33422070 10.1186/s12970-020-00403-3 PMC7796392

[16] Matias CN , Campa F , Cerullo G , et al. (2022) Bioelectrical impedance vector analysis discriminates aerobic power in futsal players: the role of body composition. *Biology (Basel)* 11, 505. 10.3390/biology11040505 PMC9025661 35453705

[17] Moroni A , Vardè C , Giustetto A , et al. (2022) Bioelectrical Impedance Vector Analysis (BIVA) for the monitoring of body composition in pregnancy. Eur J Clin Nutr 76, 604–609. 34363054 10.1038/s41430-021-00990-7

[18] Espinosa-cuevas MDLÁ , Rivas-Rodríguez L , González-Medina EC , et al. (2007) Bioimpedance vector analysis for body composition in Mexican population. Rev Investig Clínica 59, 15–24. 17569296

[19] Piccoli A , Nescolarde LD & Rosell J (2002) Conventional and vectorial analysis of bioimpedance in clinical practice. Nefrología 22, 228–238. 12123122

[20] Catapano A , Trinchese G , Cimmino F , et al. (2023) Impedance analysis to evaluate nutritional status in physiological and pathological conditions. Nutrients 15, 2264. 37242147 10.3390/nu15102264 PMC10224243

[21] Fu F , Lou J , Xi D , et al. (2020) Chest computed tomography findings of coronavirus disease 2019 (COVID-19) pneumonia. Eur Radiol 30, 5489–5498. 32435925 10.1007/s00330-020-06920-8 PMC7237879

[22] Lohman T , Roche A & Martorell R (1988) Anthropometric Standardization Reference Manual. Champaign, IL: Human Kinetics Publishers.

[23] Stewart A , Marfell-Jones M , Olds T , et al. (2011) *International Standards for Anthropometric Assessment*. Lower Hutt, New Zealand: International Society for the Advancement of Kinanthropometry.

[24] Díaz P & Fernández P (2002) Calculation of the simple size to determine prognostic factors. Cad Aten Primaria 9, 30–33.

[25] Cornejo-Pareja I , Vegas-Aguilar IM , García-Almeida JM , et al. (2022) Phase angle and standardized phase angle from bioelectrical impedance measurements as a prognostic factor for mortality at 90 days in patients with COVID-19: a longitudinal cohort study. Clin Nutr 41, 3106–3114. 33642143 10.1016/j.clnu.2021.02.017 PMC7886631

[26] Whittle J , Molinger J , MacLeod D , et al. (2020) Persistent hypermetabolism and longitudinal energy expenditure in critically ill patients with COVID-19. Crit Care 24, 581. 32988390 10.1186/s13054-020-03286-7 PMC7521195

[27] Osuna-Padilla IA , Rodríguez-Moguel NC , Rodríguez-Llamazares S , et al. (2022) Low muscle mass in COVID-19 critically-ill patients: prognostic significance and surrogate markers for assessment. Clin Nutr 41, 2910–2917. 35282986 10.1016/j.clnu.2022.02.019 PMC8886683

[28] Player EL , Morris P , Thomas T , et al. (2019) Bioelectrical impedance analysis (BIA)-derived phase angle (PA) is a practical aid to nutritional assessment in hospital in-patients. Clin Nutr 38, 1700–1706. 30170780 10.1016/j.clnu.2018.08.003

[29] Garlini LM , Alves FD , Ceretta LB , et al. (2019) Phase angle and mortality: a systematic review. Eur J Clin Nutr 73, 495–508. 29695763 10.1038/s41430-018-0159-1

[30] Alves EAS , Salazar TC , Silvino VO , et al. (2022) Association between phase angle and adverse clinical outcomes in hospitalized patients with COVID-19: a systematic review. Nutr Clin Pract 37, 1105–1116. 35932291 10.1002/ncp.10901 PMC9539244

[31] Koo BK (2022) Assessment of muscle quantity, quality and function. *J Obes Metab Syndr* 31, 9–16. 10.7570/jomes22025 PMC8987447 35318289

[32] Osuna-Padilla IA , Rodríguez-Moguel NC , Rodríguez-Llamazares S , et al. (2022) Low phase angle is associated with 60-day mortality in critically ill patients with COVID-19. J Parenteral Enteral Nutr 46, 828–835. 10.1002/jpen.2236 PMC8420520 34291834

[33] Moonen HPFX , Bos AE , Hermans AJH , et al. (2021) Bioelectric impedance body composition and phase angle in relation to 90-day adverse outcome in hospitalized COVID-19 ward and ICU patients: the prospective BIAC-19 study. *Clin Nutr ESPEN* 46, 185–192. 10.1016/j.clnesp.2021.10.010 PMC8548834 34857194

[34] Reyes-Torres CA , Flores-López A , Osuna-Padilla IA , et al. (2022) Phase angle and overhydration are associated with post-extubating dysphagia in patients with COVID-19 discharged from the ICU. Nutr Clin Pract 37, 110–116. 34617311 10.1002/ncp.10781 PMC8661566

[35] Prompetchara E , Ketloy C & Palaga T (2020) Immune responses in COVID-19 and potential vaccines: lessons learned from SARS and MERS epidemic. Asian Pac J Allergy Immunol 38, 1–9. 32105090 10.12932/AP-200220-0772

[36] Bracco D , Berger MM , Revelly JP , et al. (2000) Segmental bioelectrical impedance analysis to assess perioperative fluid changes. Crit Care Med 28, 2390–2396. 10921569 10.1097/00003246-200007000-00034

[37] Pareja IC , Vegas-Aguilar IM , Lukaski H , et al. (2022) Overhydration assessed using bioelectrical impedance vector analysis adversely affects 90-day clinical outcome among SARS-CoV2 patients: a new approach. Nutrients 14, 2726. 35807907 10.3390/nu14132726 PMC9268688

[38] Samoni S , Vigo V , Bonilla Reséndiz LI , et al. (2016) Impact of hyperhydration on the mortality risk in critically ill patients admitted in intensive care units: comparison between bioelectrical impedance vector analysis and cumulative fluid balance recording. Crit Care 20, 95. 27060079 10.1186/s13054-016-1269-6 PMC4826521

[39] Roumelioti ME , Glew RH , Khitan ZJ , et al. (2018) Fluid balance concepts in medicine: principles and practice. World J Nephrol 7, 1–28. 29359117 10.5527/wjn.v7.i1.1 PMC5760509

