# Supplementary material for: Total and segmental phase angle in a cohort of hospitalised patients with COVID-19: mortality prediction and changes throughout hospitalisation
Source: Br J Nutr. 2023 Dec 27;131(8):1397–404. doi: 10.1017/S0007114523002994 (PMC10950452; doi:10.1017/S0007114523002994)
Supplement: Estrada-Moya et al. supplementary material [file S0007114523002994sup001.pdf]

editage

Editing Certificate

This document certifies that the manuscript listed below has been edited to ensure language and grammar accuracy and is error free in these aspects. The logical presentation of ideas and the structure of the paper were also checked during the editing process. The edit was performed by professional editors at Editage, a division of Cactus Communications. The sections titled references, tables, figures and tables-legends were not edited by Editage upon the author's request.

The author's core research ideas were not altered during the editing process. Editage guarantees the quality of editing with the assumption that our suggested changes have been accepted and the edited text has not been altered without the knowledge of our editors.

MANUSCRIPT TITLE

TOTAL AND SEGMENTAL PHASE ANGLE IN A COHORT OF HOSPITALISED PATIENTS WITH COVID-19: MORTALITY PREDICTION AND CHANGES WITHIN A WEEK.

AUTHORS

Aurora E Serralde-Zúñiga

ISSUED ON

June 07, 2023

JOB CODE

LDROR_2

THE EDITAGE Promise QUALITY GUARANTEE

Vikas Narang

Vikas Narang
Chief Operating Officer - Editage

editage

CACTUS

Editage, a brand of Cactus Communications, offers professional English language editing and publication support services to authors engaged in over 1300 areas of research. Through its community of experienced editors, which includes doctors, engineers, published scientists, and researchers with peer review experience, Editage has successfully helped authors get published in internationally reputed journals. Authors who work with Editage are guaranteed excellent language quality and timely delivery.

GLOBAL :
+1(833) 979-0061 | request@editage.com

CHINA :
400-120-3020或021-6020-9400 |
fabiao@editage.cn

impact.science

researcher.life

lifesciences.cactusglobal.com
